# FlipGFP protease assay for evaluating *in vitro* inhibitory activity against SARS-CoV-2 M^pro^ and PL^pro^

**DOI:** 10.1016/j.xpro.2023.102323

**Published:** 2023-05-04

**Authors:** Haozhou Tan, Yanmei Hu, Jun Wang

**Affiliations:** 1 Department of Medicinal Chemistry, Ernest Mario School of Pharmacy, Rutgers, the State University of New Jersey, Piscataway, NJ 08854, USA

**Keywords:** Chemistry, Microbiology

## Abstract

FlipGFP assay characterizes the intracellular drug target engagement to M^pro^ and PL^pro^ and can be performed in the biosafety level 1/2 settings. Here, we provide the detailed protocol for the cell-based FlipGFP assay to identify and characterize SARS-CoV-2 M^pro^ and PL^pro^ inhibitors. We describe steps for cell passage and seeding, transfection, addition of compounds, and their incubation and timing. We then detail the quantification of the fluorescence signal of the assay

For complete details on the use and execution of this protocol, please refer to Ma et al.^1^

## Highlights

• Cell based assay for the characterization of SARS-CoV-2 M^pro^ and PL^pro^ inhibitors
• Predict antiviral activity of protease inhibitors at biosafety level 1/2 laboratory
• Reveal intracellular drug target engagement, cytotoxicity, and membrane permeability

## Abstract (editor highlights)

Publisher’s note: Undertaking any experimental protocol requires adherence to local institutional guidelines for laboratory safety and ethics.

## Graphical abstract

**Figure:**
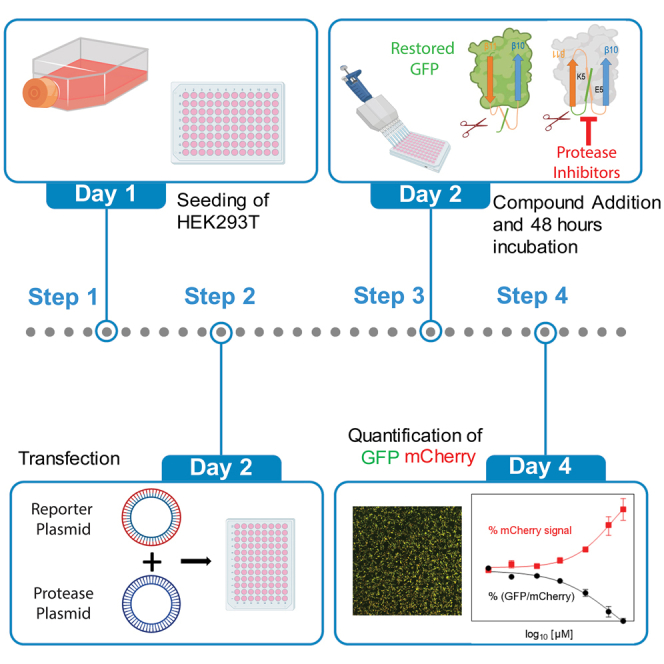

## Before you begin

### Overview

The COVID-19 pandemic caused by SARS-CoV-2 has had a devastating impact on the global economy and public health. Vaccines are mainly designed based on the viral spike protein to elicit antibody production.^2^ In parallel with vaccines, antivirals are important complements for controlling viral infection and are essential when vaccines lose potency due to mutations in the circulating viruses.^3^

The SARS-CoV-2 main protease (M^pro^, or 3CL^pro^) and the papain-like protease (PL^pro^) are high-profile antiviral drug targets.^4^^,^^5^ Both are viral cysteine proteases. M^pro^ and PL^pro^ cleave the viral polyproteins pp1a and pp1ab, producing functional units of nonstructural proteins (Nsps). The released Nsps then form the replication complex to assist the viral transcription and replication.^6^ Progress has been made in targeting M^pro^, PL^pro^, and RNA-dependent RNA polymerase (RdRp) by small molecule antivirals.^4^^,^^7^ However, with the continuous emergence of SARS-CoV-2 variants of interest (VOI) and variants of concern (VOC),^8^ vaccine efficacy is compromised. Furthermore, the current FDA-approved antivirals face several challenges: the therapeutic efficacy of remdesivir is under debate; molnupiravir has the concern of host toxicity^9^; and Paxlovid has drug-drug interaction issues.^10^ In addition, with the increasing prescription of Paxlovid, drug-resistant mutants are most likely to emerge.^11^^,^^12^ As such, additional SARS-CoV-2 M^pro^ and PL^pro^ with broad-spectrum antiviral activity, a high genetic barrier to drug resistance, and better safety profiles are needed.

During this drug discovery process, the fluorescence resonance energy transfer (FRET) assay is the gold standard *in vitro* assay for proteases. However, there is a lack of consensus on the assay condition in the scientific community, and many compounds are claimed as M^pro^ and PL^pro^ inhibitors based on the FRET assay results in the absence of reducing reagents.^1^^,^^13^^,^^14^^,^^15^ To validate the diverse M^pro^ and PL^pro^ inhibitors reported in the literature, there is a need for reliable *in vitro* assay to characterize their cellular target engagement. Herein, we introduce the FlipGFP protease assay for characterizing M^pro^ and PL^pro^ inhibitors. FlipGFP protease assay can predict the antiviral activity of M^pro^ and PL^pro^ inhibitors in biosafety level (BSL) 1/2 settings without using infectious SARS-CoV-2 virus, which requires a BSL-3 facility. FlipGFP assay has the additional advantage of ruling out compounds with cellular toxicity or poor membrane permeability.^14^^,^^16^

**Figure 1:**
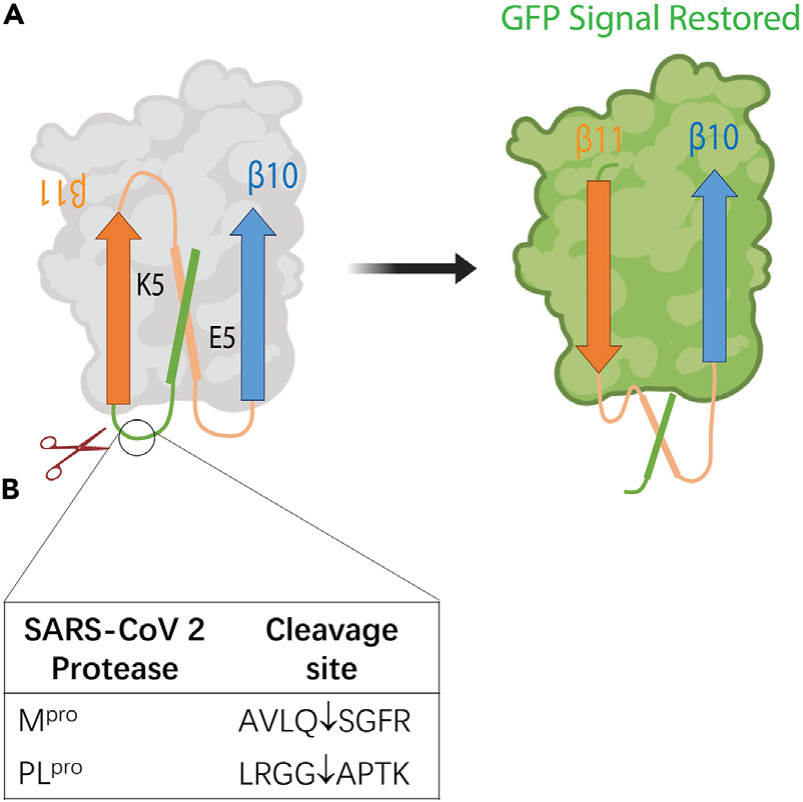
Graphic representation of the mechanism of FlipGFP assay (A) The principle of FlipGFP protease assay. When the linker between K5 and β11 is cleaved by the protease, β11 becomes antiparallel to β10, which restores the GFP signal. (B) The linker between β11 and K5 contains the SARS-CoV 2 M^pro^ or PL^pro^ substrate sequence.

In the FlipGFP protease assay, HEK-293T cells are transfected with two plasmids: one expressing the protease (M^pro^ or PL^pro^) and another expressing reporter flipped green fluorescence protein (FlipGFP) (Figure 1). The reporter FlipGFP is initially in an inactive conformation. Upon protease digestion, a conformational change in the reporter FlipGFP leads to restoring the GFP signal. Whereas in the presence of protease inhibitors, GFP signal restoration is inhibited.

We have been using FlipGFP protease assay to characterize and validate M^pro^ and PL^pro^ inhibitors in the cellular context.^1^^,^^13^^,^^14^^,^^15^^,^^17^^,^^18^ In this study, we describe the detailed protocols of the FlipGFP protease assay. The plasmid construction, HEK293T cell transfection, and incubation time have been optimized. The assay has been validated with the M^pro^ inhibitors GC376, nirmatrelvir, and the PL^pro^ inhibitor GRL0617.

### Protease expression plasmid resources & construction

This section describes the resources and construction of M^pro^ & PL^pro^ expression plasmid. For the construction of M^pro^ expression plasmid, the SARS-CoV-2 M^pro^ sequence is obtained from NCBI (NCBI: YP_009725301.1) and codon optimized for mammalian cell expression. M^pro^ sequence is constructed in pLVX expression plasmid by Addgene. For the construction of PL^pro^ expression plasmid, SARS-CoV-2 PL^pro^ sequence is obtained from NCBI (NCBI: YP_009742610.1) and codon optimized for mammalian cell expression. PL^pro^ sequence is constructed into pcDNA 3.1 expression plasmid by Genescript.

### Reporter plasmid construction

**Timing: 1 month (for steps 1 to 17)**

This section describes the construction of M^pro^ FlipGFP reporter plasmid using starting plasmid obtained from Addgene. Three PCR reactions are performed to construct the reporter plasmid. PL^pro^ FlipGFP reporter plasmid is constructed using the same method with different primers in step 2.

1. Obtain starting plasmid pcDNA3-TEV-flipGFP-T2A-mCherry from Addgene^19^ (Addgene:124429).
2. Design and synthesize the following primers from Integrated DNA Technologies (IDT).

**Table:** 

Primer name	Primer sequence
F1	GCA GAG CTC TCT GGC TAA CTA GAG
R2	TGG CAA GCT TTG GGC CAG GAT TCT C
F2-M^pro^	GCC GTG CTG CAG AGC GGC TTC AGG AAG GTG TCC GCC CTG AAG GAA AAA GT
R1-M^pro^	CCT GAA GCC GCT CTG CAG CAC GGC TGA TGC ATC GGT AAT GCC AGC CGC
F2- PL^pro^	CTG CGA GGC GGC GCT CCC ACC AAG AAG GTG TCC GCC CTG AAG GAA AAA GT
R1- PL^pro^	CTT GGT GGG AGC GCC GCC TCG CAG TGA TGC ATC GGT AAT GCC AGC CGC

***Note:*** The TEV cutting site on the starting plasmid is replaced by M^pro^(AVLQSGFR) or PL^pro^(LRGGAPTK) substrate motif using overlapping PCRs.

3. Perform PCR-1.

**Table:** PCR-1 reaction master mix

Reagent	Amount
DNA template Starting plasmid pcDNA3-TEV-flipGFP-T2A-mCherry	30–50 ng
Primer F1	1.5 μL of 10 μM stock
Primer R1-M^pro^	1.5 μL of 10 μM stock
Platinum™ SuperFi™ PCR Master Mix	25 μL
ddH_2_O	Fill to 50 μL

**Table:** PCR-1 cycling conditions

Steps	Temperature	Time	Cycles
Initial Denaturation	98°C	30 s	1
Denaturation	98°C	30 s	35 cycles
Annealing	55°C	30 s	
Extension	72°C	1 min	
Final extension	72°C	1 min	1
Hold	4°C	forever

4. Perform PCR-2.

**Table:** PCR-2 reaction master mix

Reagent	Amount
DNA template Starting plasmid pcDNA3-TEV-flipGFP-T2A-mCherry	30–50 ng
Primer F2-M^pro^	1.5 μL of 10 μM stock
Primer R2	1.5 μL of 10 μM stock
Platinum™ SuperFi™ PCR Master Mix	25 μL
ddH_2_O	Fill to 50 μL

**Table:** PCR-2 cycling conditions

Steps	Temperature	Time	Cycles
Initial Denaturation	98°C	30 s	1
Denaturation	98°C	30 s	35 cycles
Annealing	55°C	30 s	
Extension	72°C	1 min	
Final extension	72°C	1 min	1
Hold	4°C	Forever

5. Separate the PCR product of PCR-1 (and PCR-2) using 1% agarose gel electrophoresis. The size of the PCR-1 product is 381 bp, and 199 bp for the PCR-2 product.
6. Collect the gel containing PCR product, and perform purification using Wizard SV Gel and PCR clean-up System.
7. Perform PCR-3.

**Table:** PCR-3 reaction master mix

Reagent	Amount
DNA template 1: Purified PCR-1 product	30 ng
DNA template 2: Purified PCR-2 product	30 ng
Primer F1	1.5 μL of 10 μM stock
Primer R2	1.5 μL of 10 μM stock
Platinum™ SuperFi™ PCR Master Mix	25 μL
ddH_2_O	Fill to 50 μL

**Table:** PCR-3 cycling conditions

Steps	Temperature	Time	Cycles
Initial Denaturation	98°C	30 s	1
Denaturation	98°C	30 s	35 cycles
Annealing	55°C	30 s	
Extension	72°C	1 min	
Final extension	72°C	1 min	1
Hold	4°C	Forever

8. Separate the PCR product of PCR-3 using 1% agarose gel electrophoresis. The size of the PCR-3 product is 556 bp.
9. Collect the gel containing PCR-3 product, and perform purification using Wizard SV Gel and PCR clean-up System.
10. Perform endonuclease digestion for starting plasmid pcDNA3-TEV-flipGFP-T2A-mCherry.
  a. Add 1μL of endonuclease *SacI* and *HindIII.*
  b. Mix well and briefly spin down.
  c. Incubate at 37°C for 2 h.
  d. Heat-inactivate the endonuclease digestion by placing the sample on a heat block at 80°C for 20 min.
11. Separate the digested starting plasmid pcDNA3-TEV-flipGFP-T2A-mCherry using 1% agarose gel electrophoresis. The size is ∼7000 bp.
12. Collect the gel containing digested starting plasmid pcDNA3-TEV-flipGFP-T2A-mCherry, perform purification using Wizard SV Gel and PCR clean-up System.
13. Perform endonuclease digestion for purified PCR-3 product.
  a. Add 1μL of *SacI* and *HindIII* to the PCR-3 product.
  b. Mix well and briefly spin down.
  c. Incubate at 37°C for 2 h.
  d. Heat-inactivate the endonuclease digestion by placing the sample on a heat block at 80°C for 20 min.
14. Ligate the digested vector from step 10 and digested PCR-3 fragment from step 13 using T4-ligase at room temperature (∼25°C) for 4 h.

**Table:** Ligation reaction

Reagent	Amount
Vector	75 ng
Insert	50 ng
T4 Ligase	1 μL
10× Buffer	2 μL
ddH_2_O	Fill to 20 μL

15. Add 5 μL of ligation product to 60 μL of Top10 competent cells for heat-shock transformation.
16. Select ∼6 single colonies to amplify plasmid using the ZymoPure plasmid extraction kit.
17. Confirm the constructed reporter plasmid using sanger sequencing.

## Key resources table

**Table:** 

REAGENT or RESOURCE	SOURCE	IDENTIFIER
**Chemicals, peptides, and recombinant proteins**		

Trypsin-EDTA, 0.25% 1×, phenol red	Genesee	Cat#25-510
PBS (10×), pH 7.4	Thermo Fisher Scientific	Cat# 70011-069
Dulbecco’s modified Eagle medium (DMEM) with 4.5 g/L glucose, L-glutamine, sodium pyruvate	CORNING	Cat#10-013-CV
FBS	Gibco	Cat#26140-095
Nirmatrelvir (PF-07321332)	Millipore Sigma	Cat#SML3313
GRL0617	Millipore Sigma	Cat# SML2961
Penicillin-Streptomycin (P/S) 100× solution	Genesee	Cat#25-512
Agarose	Sigma Aldrich	Cat#A4718-25G
SacI-HF®	New England Biolabs	Cat#R3156S
HindIII-HF®	New England Biolabs	Cat#R3104S
rCutSmart™ Buffer	New England Biolabs	Cat#B6004S
T4 DNA Ligase	New England Biolabs	Cat#M0202S
T4 DNA Ligase Reaction Buffer	New England Biolabs	Cat#B0202S
LB Lennox Broth	IBI Scientific	Cat#IB49113
Opti-MEM I reduced serum media	Thermo Fisher Scientific	Cat#31985070

**Critical commercial assays**		

ZymoPURE II Plasmid Miniprep Kit	ZYMO Research	Cat#D4201
Wizard® SV Gel and PCR Clean-Up System	Promega	Cat#A9281
TransIT-293 transfection reagent	Mirus Bio	Cat# MIR 2705
TransIT-LT1 Transfection Reagent	Mirus Bio	Cat# MIR 2300
Platinum™ SuperFi II PCR Master Mix	Thermo Fisher Scientific	Cat#12368010

**Experimental models: Cell lines**		

HEK-293T-hACE2 cell line (Within 25 Subculture passages)	BEI Resources	Cat#NR-52511

**Oligonucleotides**		

F1 primer sequence	Integrated DNA Technologies	GCA GAG CTC TCT GGC TAA CTA GAG
R2 primer sequence	Integrated DNA Technologies	TGG CAA GCT TTG GGC CAG GAT TCT C
F2-M^pro^ primer sequence	Integrated DNA Technologies	GCC GTG CTG CAG AGC GGC TTC AGG AAG GTG TCC GCC CTG AAG GAA AAA GT
R1-M^pro^ primer sequence	Integrated DNA Technologies	CCT GAA GCC GCT CTG CAG CAC GGC TGA TGC ATC GGT AAT GCC AGC CGC
F2- PL^pro^ primer sequence	Integrated DNA Technologies	CTG CGA GGC GGC GCT CCC ACC AAG AAG GTG TCC GCC CTG AAG GAA AAA GT
R1- PL^pro^ primer sequence	Integrated DNA Technologies	CTT GGT GGG AGC GCC GCC TCG CAG TGA TGC ATC GGT AAT GCC AGC CGC
Reporter plasmid sequencing primer	Integrated DNA Technologies	TAA TAC GAC TCA CTA TAG GG

**Recombinant DNA**		

FlipGFP starting plasmid^19^^,^^20^ pcDNA3-TEV-flipGFP-T2A-mCherry	Addgene	Cat#124429
pLVX-EF1alpha-SARS-CoV-2-nsp5-2×Strep-IRES-Puro (M^pro^ expression plasmid)	Addgene	Cat#141370
pcDNA 3.1-SARS-CoV-2 PL^pro^ (PL^pro^ expression plasmid)	GeneScript	Customized Plasmid Synthesis

**Software and algorithms**		

SoftMax Pro 7.1	Molecular Devices	https://www.moleculardevices.com
Prism 8.0	GraphPad	https://www.graphpad.com

**Other**		

SpectraMax® iD3 Microplate Reader	Molecular Devices	SpectraMax® iD3
Flat-bottom 96 well cell culture microplates	Greiner	Cat#655090
Cell culture incubators, humidified, 5% CO_2_/95% air, with temperatures set up at 37°C	Eppendorf	Galaxy® 170 R
Thermal Cycler	MJ Research	Cat#PTC-200
Dry block heaters	VWR, Avantor	Cat# 75838-292
Electrophoresis Gel System	VWR, Avantor	Cat#490001-260
Electrophoresis power supply	Bio-Rad	Cat#1645070
High-Performance UV Transilluminator	UVP®	Cat# UVP-97-0246-01
Inverted microscope Olympus CKX53	Thermo Fisher Scientific	Cat#NC1991101
Microtiter plate shaker	Fisher Scientific	Cat#88-861-023
PIPETMAN Classic P1000	Genesee	Cat#37-100P1K
PIPETMAN Classic P200	Genesee	Cat#37-100P200
PIPETMAN Classic P100	Genesee	Cat#37-100P100
PIPETMAN Classic P20	Genesee	Cat#37-100P20
PIPETMAN Classic P10	Genesee	Cat#37-100P10
Vortex Mixer	Fisher Scientific	Cat#02-215-414
Centrifuge	Beckman	Cat#A99465
Sartorius™ BiohitTM Picus™ NxT Electronic Pipettes, 50-1,200 μL, 12 Channels	Sartorius™	Cat#LH745491
Sartorius™ BiohitTM Picus™ NxT Electronic Pipettes, 0.2–10 μL, 12 Channels	Sartorius™	Cat#LH745421
HandE-Vac Handheld Aspirating System	Argos Technologies™	Cat#10-987-042
Mastercycler® nexus - PCR Thermal Cycler	Eppendorf	Cat#6331000025
Reagent reservoir, sterile	VWR	Cat#89094-662
96-well storage plate	Thermo Scientific	Cat#AB-1058
Conical centrifuge tubes, sterile, polypropylene, 50 mL, 15 mL	Genesee	Cat#25-108 Cat#25-106
Serological pipets, sterile, 10 mL, 25 mL	Genesee	Cat#25-104 Cat#25-106
Pipette tips, low binding, 1000 μL, 200 μL, 10 μL	Genesee	Cat#24–160R Cat#24–150R Cat#24–121R
Tissue culture treated flasks, 600 mL, 250 mL	Genesee	Cat#25-211 Cat#25-209
1.7 mL DNase/RNase-free tubes	Genesee	Cat#25-282

***Note:*** For fluorescence signal quantification, the plate reader should be capable of quantifying and imaging green fluorescent protein and mCherry signals.

## Materials and equipment

**Table:** 10× PBS for HEK293T cell culture

Reagent	Final concentration	Amount
NaCl	80 mg/mL	400 g
KCl	2 mg/mL	10 g
KH_2_PO_4_	2.4 mg/mLc	12 g
Na_2_HPO^4^	14.4 mg/mL	72 g
ddH_2_O	N/A	Fill to 5 L
**Total**	**N/A**	**5 L**

***Note:*** To dilute 10× PBS to 1×, add 100 mL of 10× PBS to 900 mL of ddH_2_O with complete mixing. Autoclave to sterilize prior to cell culture applications.

**Table:** Complete Culture medium for HEK293T cell

Reagent	Final concentration	Amount
Dulbecco’s modified Eagle medium (DMEM) with 4.5 g/L glucose, L-glutamine, sodium pyruvate	N/A	445 mL
FBS	10%	50 mL
Penicillin-Streptomycin	100 U/mL	5 mL of concentrate
**Total**	**N/A**	**500 mL**
Complete medium can be stored at 4°C for 6 months.

**CRITICAL:** Prepare complete medium under aseptic conditions in the biosafety cabinet.

## Step-by-step method details

In the FlipGFP protease assay, two plasmids are transfected to HEK-239T cells. One expressing the protease (M^pro^ or PL^pro^) is denoted as the protease plasmid. The other expressing the reporter FlipGFP is denoted as the reporter plasmid. The reporter plasmid encodes three components, GFP β1-9, β10-11, and the mCherry (Figure 1). The β10-11 is engineered in the parallel orientation via the K5/E5 coiled coil. This orientation blocks its association with β1-9. Expression of the protease plasmid produces the M^pro^ or PL^pro^, which cleaves the M^pro^ or PL^pro^ substrate linker between K5 and β11. The cleavage shifts β10-11 into the antiparallel configuration, which can associate with β1-9 and thus restore the GFP signal. The mCherry is included to normalize the transfection efficacy and indicate the cytotoxicity of testing compounds. The assay quantifies the fluorescence signal of GFP and mCherry. The ratio of GFP to mCherry correlates with the M^pro^ or PL^pro^ enzymatic activity. Effective inhibitors have been shown to decrease the value of GFP/mCherry ratio in a dose-dependent manner.^14^

### Cell passage & seeding

**Timing: 1 h**

**Figure 2:**
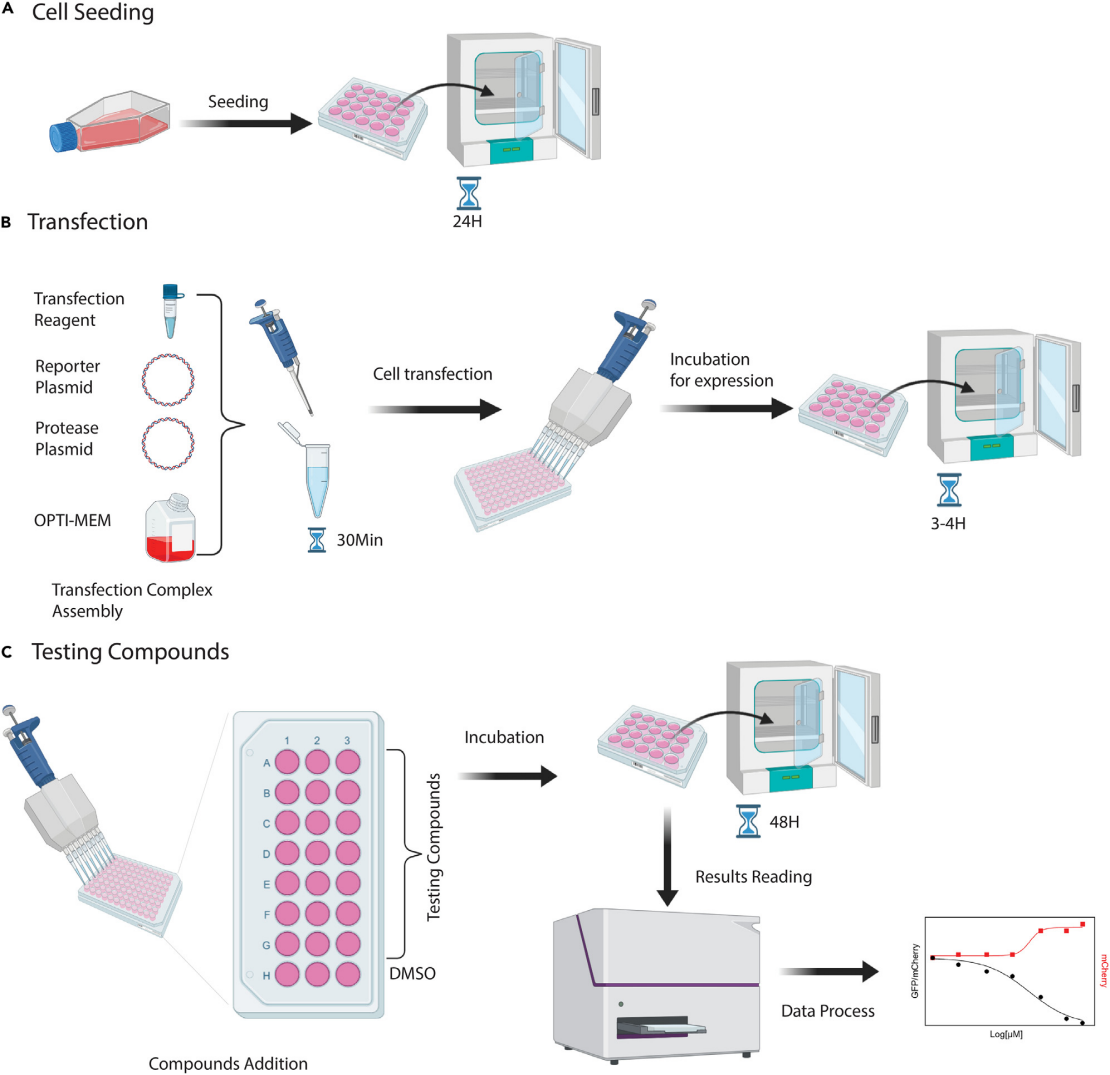
Schematic diagram of the FLIPGFP protease assay (A) Cell seeding and incubation for cell growth. (B) Steps for plasmid transfection and expression. (C) Compounds testing and data analysis.

This section describes the subculturing and seeding of the HEK293T cell line (Figure 2A).

***Note:*** all procedures should be performed under aseptic conditions in the biosafety cabinet.

1. For cell culturing in the T75 flask, perform cell passage when the confluency reaches 95–98% (95–98% surface area of culture flask is covered by HEK293T cells).
  a. aspirate cell culture medium.
  b. Rinse cells with 10 mL (for T75 culture flask) of DPBS with gentle swirling to remove the remaining fetal bovine serum, which inhibits trypsin.
  c. Repeat the PBS rinse once.
2. Add 3 mL trypsin-EDTA solution to the cell monolayer in the T75 culture flask. Agitate gently and incubate the flask at room temperature(20–25°C) for 1 min or until the cell just begin to detach.
3. Collect the detached cells.
  a. Gently tap the flask to detach the cell.
  b. Add 10 mL prewarmed (to room temperature) complete culture medium containing DMEM (with 4.5 g/L glucose, L-glutamine, and sodium pyruvate), 10% FBS, and 1% penicillin-streptomycin to the flask.
  c. Gently disperse the medium with pipetting using a serological pipette over the cell monolayer to recover 95% of the cells.
  d. Mix the cell suspension by pipetting up and down.
4. Transfer cell suspension to a 15 mL centrifuge tube and centrifuge at 500 × *g* for 5 min at room temperature (20°C–30°C).
5. Aspirate the supernatant and resuspend the cell pellet with 10 mL of complete culture medium.
6. Seed the cell to a 96-well plate with 100 μL per well and a 0.8–3.0 × 10^5^ cells/mL density.
7. Incubate the flask in a cell culture incubator (Humidified, 5% CO_2_/95% air, 37°C) overnight (16–18 h) (Figure 2A).

***Note:*** HEK293T cells are suitable for FlipGFP assay within 25 passages. The transfection efficiency is reduced beyond 25 passages.

**CRITICAL:** it is essential to ensure cells reach 70%–80% of confluence at the time of transfection to achieve the most efficient transfection*.* More than 95% confluence may result in low efficiency of transfection.

### Transfection

**Timing:** 2 h (for steps 25 to 31)

HEK293T cells are transfected with two plasmids: one expressing the protease and another expressing the FlipGFP reporter (Figure 2B).

This section describes the plasmid transfection procedure in the FlipGFP assay.

8. On the second day after cell seeding, check the cell confluency in the T75 flask using a phase-contrast inverted microscope. 70–80% confluence gives the most efficient transfection.
9. Dilute GFP reporter plasmid and protease plasmid to 500 ng/μL using autoclaved nuclease-free water.
10. To assemble the transfection complex, mix 9 μL of Opti-MEM, 0.1 μL of 500 ng/μL reporter plasmid, 0.1 μL of 500 ng/μL protease plasmid, and 0.3 μL of transIT-293 for each well of a 96-well plate (Figure 2B).

***Note:*** master mix for multiple wells is recommended.

11. Gently vortex the transfection complex and incubate at room temperature for 30 min.
12. Add 9.5 μL of transfection complex to each well of the 96-well plate, such that 50 ng of GFP reporter plasmid and 50ng of protease plasmid are aliquoted to each well.
13. Mix the transfection complex in each well by shaking the plate on a shaker for 5 min.
14. Incubate the plate in a cell culture incubator (humidified, 5% CO_2_/95% air, 37°C) for transfection and protein expression. Incubate for 3 h.

**Figure 3:**
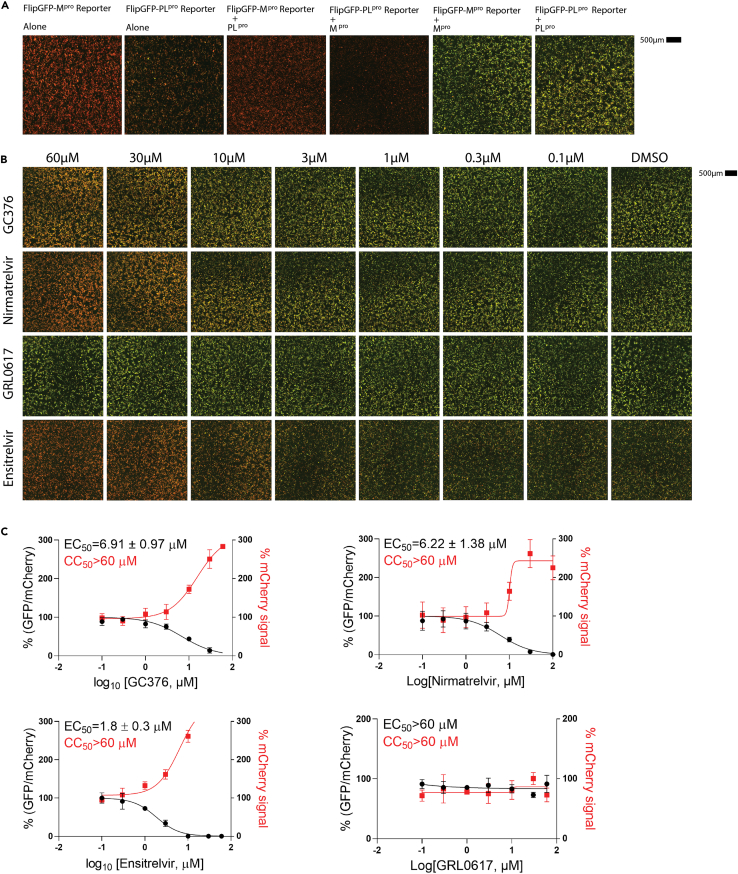
Assay Characterization and FlipGFP assay for SARS-CoV-2 Mpro inhibitors (A) Images of FlipGFP assay validation. Scale bar represents 500 micrometers. (B) Images of FlipGFP M^pro^ assay for GC376, Nirmatrelvir, and Ensitrelvir. GRL0617 was included as a negative control. The images are representatives of three individual repeats. Scale bar represents 500 micrometers. (C) The dose-response curve of the GFP/mCherry fluorescence ratio and the EC50 for GC376, Nirmatrelvir, Ensitrelvir, and GRL0617 in FlipGFP M^pro^ assay. The mCherry signal was used to normalize M^pro^ expression level and characterize compound toxicity. Curve fittings are obtained in GraphPad Prism 8. Error bars represent the standard deviation of the data.

***Note:*** To validate the FlipGFP assay, control experiments with matching or mismatching protease-FlipGFP reporter pairs need to be performed (Figure 3A). In addition, control compounds should be tested. Positive control (a compound with potent protease inhibitory activity and without cytotoxicity) should show dose-dependent inhibition of the GFP signal with a consistent EC_50_ value. Negative control (a compound without protease inhibitory activity and cytotoxicity) should show a constant GFP signal in different concentrations. The positive and negative control groups should show a consistent mCherry signal to indicate successful transfection and expression.

### Testing compounds

**Timing: 10 min**

This section describes the FlipGFP assay procedures of compound addition.

15. Prior to compound addition, the testing M^pro^ or PL^pro^ inhibitors and control compounds (GC376 and GRL0617) should be diluted in DMSO with a concentration gradient of 6, 3, 1, 0.3, 0.1, 0.03, 0.01 mM.

***Note:*** avoid using wells on the edge of the compound testing, including the control well of the DMSO-treated group. Evaporation of media in the edge wells may affect results.

16. After 3 h of incubation, add 1 μL of compound to the well (final concentration 60, 30, 10, 3, 1, 0.3, 0.1 μM), and mix well immediately by shaking the plate on a shaker with moderate speed for 5–8 min. (Figure 2C).

***Note:*** A concentration of up to 1% (v/v) DMSO can be used without a significant cytotoxic effect on HEK293T cells.

17. Incubate the 96-well plate for 48 h at 37°C, 5% CO_2_/95% air.

**CRITICAL:** Addition of compound dissolved in DMSO can damage the cell layer if not mixed well immediately. Compound should be added to the edge of the well and shaken immediately to ensure even mixing.

### Quantification

**Timing: 30 min**

This section describes the quantification of the fluorescence signal of the assay.

**Figure 4:**
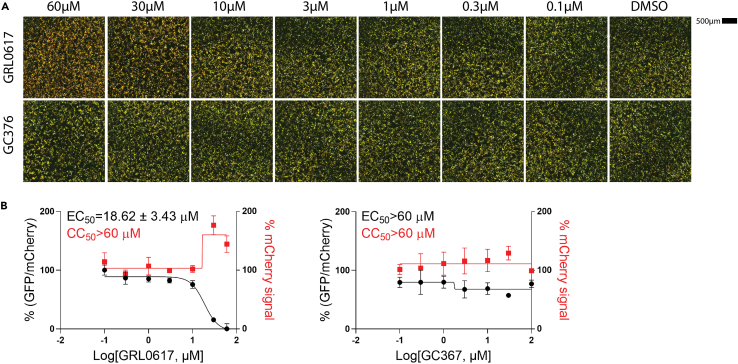
FlipGFP assay for SARS-CoV-2 PL^pro^ inhibitors (A) Images of FlipGFP PL^pro^ assay for GRL0617. GC376 was included as a negative control. The images are representatives of three individual repeats. Scale bar represents 500 micrometers. (B) The dose-response curve of the GFP/mCherry fluorescence ratio and the EC50 for GRL0617 and GC376 in FlipGFP PL^pro^ assay. The mCherry signal was used to normalize PL^pro^ expression level and characterize compound toxicity. Curve fittings are obtained in GraphPad Prism 8. Error bars represent the standard deviation of the data.

***Note:*** Multiple point scan of each well is highly recommended. For detailed data analysis, please refer to the quantification and statistical analysis section.

18. After 48 h of incubation, quantify GFP and mCherry signal in a plate reader (Figures 3A, 3B, and 4A).
  a. Set excitation at 485 nm, and emission at 530 nm for green GFP fluorescence quantification.
  b. Set excitation at 580 nm and emission at 630 nm for red mCherry fluorescence quantification.

## Expected outcomes

To calibrate the specificity of the FlipGFP assay, cells are transfected with either the FlipGFP reporter plasmid alone or with the matching or mismatching protease plasmid (Figure 3A). Transfection of cells with FlipGFP M^pro^ or PL^pro^ reporter plasmid alone should only produce mCherry signal but not GFP signal (Figure 3A first two columns). Similarly, cells transfected with mismatching pairs (FlipGFP M^pro^ reporter plasmid + PL^pro^ plasmid; FlipGFP PL^pro^ reporter plasmid + M^pro^ plasmid) should also only produce mCherry signal but not GFP signal (Figure 3A third and fourth columns). Only cells transfected with the matching pairs (FlipGFP M^pro^ reporter plasmid + M^pro^ plasmid; FlipGFP PL^pro^ reporter plasmid + PL^pro^ plasmid) produce both mCherry and GFP signals (Figure 3A last two columns).

In the presence of a potent protease inhibitor, dose-dependent inhibition of GFP signal should be observed (Figure 3B first, second, fourth rows, 4A top row). For the negative control compound, GFP signal is constant with all testing concentrations (Figure 3B third row, 4A bottom row).

## Quantification and statistical analysis

This section uses FlipGFP PL^pro^ assay as an example for data analysis. The readout of FlipGFP assay includes the GFP readings of testing compounds and the control compound at different concentrations, the mCherry readings of the testing compounds and the control compound at different concentrations.

**Table:** 

GRL0617 concentration μM	GFP readout		
60	359036	337656	339321
30	469112	431043	414790
10	479215	450564	371494
3	434613	448383	426458
1	481669	439163	391477
0.3	477668	462277	402752
0	483514	455049	455949

GRL0617 concentration μM	mCherry readout		
Background	6120	7311	5894
60	328914	322504	327344
30	361845	346443	323188
10	295941	306757	241794
3	274094	269048	279229
1	278434	284904	256545
0.3	297818	293431	239296
0	276082	290705	273383

The ratio of GFP readout to mCherry readout (GFP/mCherry) is calculated and plotted against log-scale concentration to determine the half maximal effective concentration (EC_50_).

$$\frac{GFP\;Signal\;Readout}{mCherry\;Signal\;Readout}$$

The mCherry readout is plotted against log-scale concentration to determine the half maximal cytotoxic concentration (CC_50_).

Both GFP/mCherry ratio and mCherry alone should be normalized prior to EC_50_ and CC_50_ plotting.

For the normalization of EC_50_ analysis, the GFP/mCherry value of the DMSO treated group is defined as 100%. The GFP/mCherry value of the control compound at 60 μM (highest concentration) is defined as the cut-off of 0% for the control compound itself and all testing compounds. A control compound should be included in each experiment. In CC_50_ analysis, the mCherry readout of the DMSO-treated group is defined as 100%. The mCherry readout of the non-transfected group is the background of the assay and is defined as 0%.

**Table:** 

GRL0617 concentration μM	GFP/mCherry value		
60	1.09	1.04	1.03
30	1.29	1.24	1.28
10	1.61	1.46	1.53
3	1.58	1.66	1.52
1	1.72	1.54	1.52
0.3	1.6	1.57	1.68
0	1.75	1.56	1.66

GRL0617 concentration μM	Normalized GFP/mCherry (%GFP/mCherry)		
60	5.5	-1.9	-3.6
30	39.5	30.8	37.3
10	93.0	68.0	79.3
3	87.4	100.8	77.7
1	111.3	80.1	77.5
0.3	90.4	85.7	103.6
0	114.9	84.1	101.0

GRL0617 concentration μM	Normalized mCherry (%mCherry)		
Background	-0.1	0.3	-0.2
60	117.9	115.5	117.3
30	129.9	124.3	115.8
10	105.8	109.8	86.0
3	97.8	96.0	99.7
1	99.4	101.8	91.4
0.3	106.5	104.9	85.1
0	98.5	103.9	97.6

In GraphPad Prism 8, create ‘New table & graph’, select ‘XY’ on ‘options’, and enter 3 replicates for Y. Transform compound concentration to log and input in column X. Three replicates of GFP/mCherry values at each concentration of compound are input in column Y. Click the ‘Analyze’ button, and select “Normalize’. For 0%, enter the mean of the 60 μM control compound treated group. For 100%, enter the mean of the DMSO-treated group. The EC_50_ of testing compound is determined by fitting the curves with nonlinear regression using log (inhibitor) vs. response with variable slopes (Figure 3C and 4B).

***Note:*** GFP/mCherry value will not be dose-dependent for compounds with cytotoxicity at high concentrations. This is reflected by the decrease of mCherry signal at higher drug concentrations due to cytotoxicity. In this situation, discard the GFP/mCherry values at toxic concentrations and re-plot the lower concentrations.

**Table:** 

Compound μM	GFP/mCherry value		
60 (Toxic concentration)	1.4	1.4	1.5
30	0.5	0.5	0.6
10	0.7	0.7	0.8
3	1.1	1.3	1.3
1	1.4	1.6	1.7
0.3	1.6	1.6	1.7
0	1.5	1.7	1.7

## Limitations

The FlipGFP assay has the advantage of predicting the antiviral activity in the cellular context in the biosafety level 1/2 facilities. Compared to FRET enzymatic assay, FlipGFP assay also rule out compounds with poor membrane permeability and cellular cytotoxicity.

Nevertheless, FlipGFP assay has several limitations. Since it is a cell-based assay requiring the delicate operation of transfection, it is not suitable for high-throughput screening (HTS). Furthermore, compounds with fluorescence interference properties may give false positive results. Therefore, FRET and binding assays must be performed to validate the results.

An efficient antiviral drug discovery pipeline should start with FRET-based high-throughput screening. Next, FlipGFP assay can be applied to characterize their cellular protease inhibitory activity and rule out compounds with poor membrane permeability or cytotoxic. With these assays, potent candidates can be efficiently identified for the next step of antiviral assay and *in vivo* animal model studies.

## Troubleshooting

### Problem 1

No fluorescence signals.

### Potential solutions

Usually caused by failure of transfection and protein expression. Double-check the sequence of both protease plasmid and FlipGFP reporter plasmid by sanger sequencing (Step 17 in step-by-step method details). Confirm the sequence of the digestible linker on the reporter plasmid (Sequencing primer provided in key resources table).

Double-check the cell line for contamination when the plasmid sequence is confirmed. Finally, test the control compound to verify if the assay is working.

Cytotoxicity is also a cause for no signal, and this issue usually occurs when compound concentration is above the toxic threshold. Concentrations below the cytotoxic threshold should produce GFP and mCherry signals if the transfection and expression are successful.

### Problem 2

Low fluorescence signal.

### Potential solutions

Low fluorescence signal is usually caused by inefficient transfection. Check and adjust the cell confluence at the time of transfection (Step 8 in step-by-step method details). More than 95% of confluence may result in low efficiency of transfection and hence result in low fluorescence signal. The optimal confluency for transfection is 70–80%. Besides, subculturing HEK-293T cell for more than 25 passages also result in reduced transfection efficiency and low signal. If the cell line is more than 25 subcultures, discard the cell, and use the HEK-293T cell lines with fewer subculture cycles.

### Problem 3

Cell detachment.

### Potential solutions

Cell detachment is usually caused by contamination and improper handling. For contamination, discard the cell and initiate new HEK-293T from stock. After two cycles of subculturing, the newly initiated cell line is ready for assay. To avoid HEK-293T detachment, transfection complex and compound solution should be added gently to the edge of the well of the culture plate.

### Problem 4

Failure of control compounds.

### Potential solutions

GC376 is used as the positive control for FlipGFP M^pro^ assay, and GRL0617 is used as a positive control for FlipGFP PL^pro^ assay. When the control compounds fail to show dose-dependent inhibition, first discard the Opti-MEM. Use unexpired, clean Opti-MEM™ I Reduced Serum Medium. Then double-check if the cell line is healthy, not contaminated, and within 25 subcultures. Finally, double-check and repeat the compound dilution and transfection complex to ensure the correct compound concentration, plasmid concentration, and transfection reagent volume (Step 10 in step-by-step method details).

### Problem 5

Large variations.

### Potential solutions

Large variations are typically caused by cell detachment, insufficient mixing, and usage of edge wells of the culture plate. For cell detachment, please refer to the solutions in problem 3. For insufficient mixing of transfection complex and compound, shake the plate on a shaker immediately after addition at 100 rpm for 8 min (Steps 13 and 16 in step-by-step method details). Finally, avoid using the edge wells on the 96-well plate, as solutions in these wells have evaporation issues.

## Resource availability

### Lead contact

Haozhou Tan: ht359@pharmacy.rutgers.edu.

### Materials availability

This study did not generate new reagents.

## Data Availability

This study did not use any database and code.
